# Editorial: Prioritizing pleasure in reproductive and maternal health to address obstetric violence

**DOI:** 10.3389/fgwh.2026.1857244

**Published:** 2026-07-30

**Authors:** Kaveri Mayra, Nicole Miriam Daniels, Malena Correa

**Affiliations:** 1 Department of Family Practice, The University of British Columbia, Vancouver, BC, Canada; 2 Center for Social Science Research, University of Cape Town, Rondebosch, South Africa; 3 Department of Community Health and Social Sciences, Universidad Nacional de Jose Clemente Paz, José C. Paz, Argentina

**Keywords:** childbirth, maternal health, obstetric violence, pleasure in labour and birth, reproductive justice

What a radical idea and gratifying experience it is to center a special issue on women's pleasure and the pleasure of trans, non-binary, and gender non-conforming people. Particularly at the backdrop of political warfare in different parts of the world, this Research Topic of papers provides an antidote to such violence. Our Research Topic “*prioritizing pleasure in reproductive and maternal health to address obstetric violence”* presents the seemingly contradictory idea that in the face of violent toxicity it is more important than ever to orientate towards pleasure. The reasons are multi-facetted but hinge on a central theme, which is that pleasure, as a conduit for relational connection, shared purpose and meaning, restores dignity, humanity and respect. This is pleasure activism (1) for the purpose of ending obstetric violence (OV).

In this issue, we expand the discourse on OV, a form of gender-based violence that violates fundamental human rights, occurs in obstetric systems, and is rooted in colonial, ableist and racialized medical systems. The articles span a wide range of scholarship and approach the topic from multiple view-points. Authors take a critical feminist stance; use historical/political lenses; decolonizing arts-based research approaches; experiential and economic frameworks; and narrative, intervention-based approaches, to illuminate different entry-points into pleasure as an antidote to violence.

Pascali-Bonaro explores orgasm as the epitome of pleasure during childbirth, which is an experience too often overlooked or silenced in traditional medical settings. Presenting a framework centered on love, empowerment, and humanized perinatal care, she argues that pleasure during labor is not accidental but arises spontaneously as the result of neurophysiological systems in the body. Central to the social environment that makes birthing pleasurable is positive talk, intimacy, and relaxation, which support instinctual and hormonal pathways that sustain the body's own intelligence. Pascali-Bonaro advocates for a radical shift in practice, suggesting that pleasurable stimulation and intimacy can be used as an antidote to traumatic births. By encouraging care providers to integrate pleasure-based practices as forms of pain relief, Pascali-Bonaro who is a doula, childbirth educator and pleasure advocate, seeks to transform the birthing journey into a source of connection, satisfaction, compassion and profound bodily agency.

Botes' original research describes the historical erasure of South Africa's Black midwives, the *Voetvroue*, whose autonomous knowledge has been marginalized by centuries of colonial and racialized medical governance. Botes locates modern obstetric violence within a long history of epistemic repression, arguing that the medicalization of birth served as a tool for controlling Black bodies. Central to the decolonial model proposed by Botes is the concept of “radical care,” which redefines pleasure not as a luxury, but as a vital emotional, spiritual, and relational force. By prioritizing pleasure in both caregiving and care-seeking, Botes presents a reparative framework that resists biomedical control over birth givers and indigenous knowledge keepers of autonomous birth. Ultimately, the article argues that reclaiming birth as a sacred act is an essential obligation in our current era of upheaval, offering a decolonial and revindicative path toward reproductive justice.

Kumar et al. offer a thought experiment that seeks to move the dial beyond basic medical safety. Their commentary advocates for a paradigm shift in the economic evaluation of perinatal interventions. They introduce the measurement of the Positive Potential Value (PPV) of birth, an economic model that employs a strength-based approach to measure the “*well-being enhancing potential of a positive birth experience”.* By accounting for the capability and confidence enhancing impacts of birth, this empowered model seeks to redefine “*what good looks like”* through contextually and culturally specific lenses. The authors raise an important question “*Do economists believe that birth experiences do not matter or is there some other reason we are not counting them?”* to argue that economic evaluations must evolve to incorporate thriving; not just surviving. This shift recognizes birth not just as a medical event to be managed, but as a significant opportunity for human flourishing that is both clinically sound and experientially transformative.

Taye and Belachew's original research evaluates a multi-faceted intervention designed to reduce OV through respectful maternity care education for health care providers and maternity “*open days”* for expectant individuals in public hospitals in Southwest Ethiopia. They combine these with empowerment strategies that allow birthgivers space for questions and educate them on their rights. The trust and bond this enables between the care seeker and provider is an underrated and under-researched aspect of pleasure in the birthing environments. The significant reduction across all types of OV through their intervention suggests that mitigating systemic harm requires a holistic shift. It posits that improved maternal health outcomes depend on integrating dignity and pleasure as fundamental components of ordinary maternity care, transforming it into a site of respectful support for birth givers *and* healthcare providers.

McKenzie's original research focuses on the joy and pleasure of the controversial choice of undisturbed physiological birthing (freebirthing), which is framed as a transformative exercise of agency and reclaiming bodily autonomy, especially in states and countries that govern, control and police wombs and bodies. The author focuses specifically on a form of poetic inquiry (that involves multiple listenings to understand the participant's narrative), to understand the relational dynamics in the birthing environment that encourage, enhance and enable pleasurable birthing and the ones where it feels oppressive. The beauty of these approaches lies in presenting narratives with emotional sensitivity and compassion. The research underscores the role of alternative knowledge networks and doulas's - suggesting that storytelling from women's perspectives serves as a potent, empowering form of knowledge-making. A central finding, in the poem freebirth, is the reporting of *euphoria* as a complex mixture of pain and the unmatched pleasure that comes from confidence and trust in one's body. This work contributes to broader discussions on reproductive autonomy and how resisting medicalization allows birth to be reclaimed as a joyful journey grounded in women's endurance and agency.

Gender-based violence has long entered our bedrooms in the form of intimate partner violence and domestic violence; and has also entered birthing rooms as obstetric violence. We need something radical to reclaim birth and reclaim midwifery to address not just over medicalised obstetric practice, but also restorative justice. Reclaiming pleasure for women and queer people, whose desires are considered forbidden, is political (2). The pathway to transform birthing experiences and childbirth care is through a sexual revolution (3) and pleasure activism (1). Newer framings and understanding of love and sex (4) are required to acknowledge and account for gender, power, race, caste and sexualities and the relationships between all the intersectionalities; where women, girls and queer people's pleasure is priority. Hooks (5) offers a way to love that is healing and redemptive for people, systems and nations that is relational and based on connections and compassion. While feminist economics highlights that in boardrooms and bedrooms across the globe, the fight for gender equality remains relevant (6). These and other discourses point towards the right to pleasure in all walks of life, including when people give and assist others to give birth.

## References

[1] Brown AM. Pleasure Activism- the Politics of Feeling Good. Edinburgh: AK Press (2019).

[2] Rajasekaran S. Forbidden Desire: How the British Stole India’s Queer Pasts and Queer Futures. New Delhi: Simon and Schuster India (2025).

[3] Penny L. Sexual Revolution. Dublin: Bloomsbury Publishing (2022).

[4] Srinivasan A. The Right to sex. Dublin: Bloomsbury Publishing (2021).

[5] Hooks B. All About Love. New York: HarperCollins Publishers (2001).

[6] Low C. Femonomics: The Empowering Data Driven Guide to Making Better Decisions at Home and Work. New York: Hodder and Stoughton (2025).

